# Declining malaria parasite prevalence and trends of asymptomatic parasitaemia in a seasonal transmission setting in north-western Burkina Faso between 2000 and 2009–2012

**DOI:** 10.1186/1475-2875-12-27

**Published:** 2013-01-22

**Authors:** Carolin Geiger, Hani Kartini Agustar, Guillaume Compaoré, Boubacar Coulibaly, Ali Sié, Heiko Becher, Michael Lanzer, Thomas Jänisch

**Affiliations:** 1 Department for Infectious Diseases, Parasitology, University Hospital Heidelberg, Im Neuenheimer Feld 324, Heidelberg, 69120, Germany; 2 Centre de Recherche en Santé à Nouna, Nouna, BP02, Burkina Faso; 3 University Hospital Heidelberg, Institute for Public Health, Im Neuenheimer Feld 324, Heidelberg, 69120, Germany

**Keywords:** Malaria, Transmission, Parasite prevalence, Parasite density, *Plasmodium falciparum*, Mixed infections, Asymptomatic parasitaemia, Seasonal transmission, Clinical malaria, Burkina Faso

## Abstract

**Background:**

Malaria transmission was reported to have declined in some East African countries. However, a comparable trend has not been confirmed for West Africa. This study aims to assess the dynamics of parasite prevalence and malaria species distribution over time in an area of highly seasonal transmission in Burkina Faso. The aim was also to compare frequency of asymptomatic parasitaemia between wet and dry season by parasite density status and age group.

**Methods:**

During the years 2009–2012, six cross-sectional studies were performed in the rural village Bourasso in the Nouna Health District in north-west Burkina Faso. In subsequent rainy and dry seasons blood samples were collected to assess the parasite prevalence, species, density and clinical parameters. In total, 1,767 children and adults were examined and compared to a baseline collected in 2000.

**Results:**

The microscopical parasite prevalence (mainly P. falciparum) measured over the rainy seasons decreased significantly from 78.9% (2000) to 58.4%, 55.9% and 49.3%, respectively (2009–2011; p <0.001). The frequency of *Plasmodium malariae* infections (mono- and co-infections) decreased parallel to the overall parasite prevalence from 13.4% in 2000 to 2.1%, 4.1% and 4.7% in 2009–2011 (p <0.001). Comparing parasite-positive subjects from the rainy season *versus* dry season, the risk of fever was significantly reduced in the dry season adjusting for parasite density (grouped) and age group.

**Conclusions:**

The results of this study suggest a decline of malaria transmission over the rainy seasons between 2000 and 2009–2011 in the region of Nouna, Burkina Faso. The decreased transmission intensity was associated with lower prevalence of *P. malariae* infections (both mono-infections and co-infections). Asymptomatic parasitaemia was more frequent in the dry season even adjusting for parasite density and age group in a multivariate regression. Possible reasons for this observation include the existence of less pathogenic *Plasmodium falciparum* genotypes prevailing in the dry season, or the effect of a reduced incidence density during the dry season.

## Background

Malaria is one of the major health problems in sub-Saharan Africa (SSA) with approximately 174 million cases and 655,000 deaths per year, which is >80% of cases and >90% of malaria deaths worldwide [1]. Since the 1970s the University of Heidelberg maintains a partnership with the Centre de Recherche en Santé Nouna (CRSN) in Nouna, Burkina Faso [2]. A number of research projects have been conducted during this cooperation with an emphasis on disease control of malaria [3-6]. A health and demographic surveillance system (HDSS) was established in 1992 and has monitored a population of 78,000 at regular intervals [2].

Malaria transmission and morbidity has been reported to have declined in areas of East Africa, which is assumed to be at least partly a result of the up-scaling of interventions (e g, availability of artemisinin combination therapy (ACT) and distribution of insecticide-treated bed nets (ITNs) [7-10]. With regard to West Africa, this trend is not well documented with the exception of few hospital-based studies [11], which have their own limitations [12]. In this study, cross-sectional data from 2009–2012 were compared to a baseline survey from 2000, which was already published elsewhere [13]. The study was initially designed to monitor genotypic drug resistance mutations, which will be reported separately.

The epidemiology of asymptomatic malaria in different transmission settings is attracting increasing attention, because asymptomatic individuals are still able to produce gametocytes and therefore provide the reservoir for transmission [14,15]. The majority of malariological studies are carried out in areas of high and stable transmission and less is known about settings with marked seasonal transmission [16]. The study reports age-stratified malaria prevalence, parasite densities, mixed infections and clinical parameters in a series of population-based, cross-sectional surveys between 2000 and 2012. The surveys were carried out both in rainy and dry season in north-western Burkina Faso.

Infections with multiple *Plasmodium* species are common in malaria-endemic areas and it has been proposed that interaction between different species may influence the epidemiology and clinical presentation of malaria [17]. However, there is not much data about the interaction of these kinds of co-infection in different transmission settings and the existing data is conflicting. This study reports about the distribution of *Plasmodium* species by microscopy and PCR to monitor the variation between the different seasons over time.

## Methods

### Study area and study design

The study was conducted to evaluate the levels of anti-malarial drug resistance on a molecular basis in Bourasso village, Kossi District, Burkina Faso, approximately 30 km from the district town Nouna. The village is located within the area of the Nouna Health and Demographic Surveillance System (HDSS), which included almost 80,000 individuals under constant demographic surveillance. The HDSS population was used as the sample frame for various epidemiological and clinical studies [5,6]. The under-five mortality rate in this area has dropped from about 40 per 1,000 person-years in the mid-1990s to below 30 per 1,000 in 2007 [2]. Malaria is hyper- to holo-endemic in this area with a transmission peak at the end of the rainy season (June to October) and reduced transmission during the dry season (November to May) [3]. The dry season consists of a cold period from November to February and a very hot phase between March and May. Bourasso had 2,263 inhabitants of different ethnic groups in March 2011 who mainly live from subsistence farming and cattle keeping. A first cross-sectional survey, which was already described elsewhere [13,18,19], was carried out in October 2000 and served as a baseline for this study. Starting in 2009 until 2012 the survey was repeated both in October and April at the end of the rainy and dry season respectively.

A random list of all households in the village was generated using the data from the HDSS. All household members of the first 50–100 households were invited to participate until the target of ~250 participants was reached (inclusion criterion above six months of age). Sample size was determined with the aim of ~50 parasite positive isolates available for drug resistance testing per cycle. For the dry season an overall parasite prevalence of ~15% (no previous data available) was assumed, which later had to be updated. The assumed parasite prevalence for the rainy season was 80% as it was reported in previous studies [13].

Written informed consent was taken from all patients prior to enrolment. Anthropometry for children was performed by skilled health care personnel. Each patient was examined by a physician including recent history of drugs taken. A blood sample (maximum 5 ml) was taken by a skilled provider. Thick and thin blood smears were prepared and parasite density counted against 200 WBC [20]. One drop of blood was applied to filter paper (either GenoCard™, Hain Lifesciences, Germany or Whatman™ 3MM chromatography paper, Brentfort, UK). The filter papers were air-dried, stored in plastic bags and transported to the Parasitology Laboratory at Heidelberg University Hospital for molecular analysis. The blood smears were fixed in methanol and stained with Giemsa solution to be analysed on the spot. Treatment according to MOH guidelines was provided without charge. Patients whose blood smears were positive for *Plasmodium* parasites were treated with amodiaquine-artesunate. The blood sample was divided into blood for culture and serum for pharmacological analysis of anti-malarial drugs, then frozen and transported to Heidelberg for further experiments. Ethical approval was obtained by the ethical review board of Heidelberg University Hospital and the Institutional Review Board in Nouna.

The genomic DNA was extracted from the filter papers using the Chelex-100 method [21]. The different *Plasmodium* species were analysed by microscopy and species-specific nested-PCR [22], the positivity for *Plasmodium falciparum* was the basis for the calculated PCR prevalence.

Graphs were created with Sigma-Plot 11 (Systat Software, Chicago, USA). Statistical analysis was performed using STATA 11 program (Stata Corporation, Duxbury, USA). A t-test for proportions was used to assess the significance of differences between parasite prevalences and species distributions in dry and wet season of this study. A Mann–Whitney Rank Sum Test was carried out to compare the parasite densities excluding all zero counts. To evaluate the joint influence of season, age, and parasite density on symptomatic disease/fever a logistic regression analysis was performed.

## Results

During the first cross-sectional study in 2000 all inhabitants of the village Bourasso were included, in total 1,561 individuals [13]. In the years 2009–2012 a random subsample based on household numbers selected through the HDSS was invited to participate. Altogether 1,767 individuals were included (Table 1): 402, 256 and 219 in the rainy season in 2009, 2010 and 2011, and 362, 267 and 261 in the dry seasons 2010, 2011 and 2012. Participants were aged between six month and 90 years, albeit the median age was 13 (IQR 6–33). There were slightly more female individuals (52.8%) than males in the study group 2010–2012.

**Table 1:** **Demographic characteristics, malaria parasite prevalence (by microscopy and PCR), and proportion of symptomatic malaria infections in the study population by year (2000 *vs *2009–2012) and season**

**Rainy seasons**	**October 2000**	**October 2009**	**October 2010**	**October 2011**
Number of participants	1561	402	256	219
Median age (IQR) [years]	14 (6–30)	11 (5–28)	15 (9–37)	13 (6–33)
Age range [years]	0-90	1-89	1-90	1-74
Proportion of male participants [%]	50.5	46.5	53.1	48.4
Parasite prevalence microscopy [%] (CI95%)	78.9 (76.8-80.9)	58.4 (53.4-63.2)	55.90 (49.5-62.0)	49.3 (42.5-56.1)
Parasite prevalence PCR [%] (CI 95%)	92.0 (87.4-95.4)^*^	66.1 (61.1-70.7)^**1**^	68.8 (62.7-74.4)^**1**^	73.7 (67–79.7)^**2**^
Number of symptomatic patients [%]**		14.7	9.8	15.1
**Dry seasons**		**April 2010**	**April 2011**	**April 2012**
Number of participants		362	267	261
Median age (IQR) [years]		14 (5–34)	15 (7–33)	13 (7–37)
Age range [years]		1-78	0-77	1-78
Proportion of male participants [%]		44.8	44.9	45.6
Parasite prevalence microscopy [%] (CI95%)		30.1 (25.4-35.1)	29.6 (24.1-35.5)	32.6 (26.9-38.6)
Parasite prevalence PCR [%] (CI95%)		39.4 (33.6-45.4)^**1**^	39.3 (33.4-45.5)^**1**^	34.6 (28.8-40.7) ^2^
Number of symptomatic patients [%]**		4.6	1.3	3.8
**Combined estimates**	**Combined rainy seasons 2009-2011**	**Combined data dry seasons 2010-2012**		
Number of participants	877	890		
Median age (IQR) [years]	13 (6–31)	14 (6–35)		
Age range [years]	0-90	0-78		
Proportion of male participants [%]	48.9	45.0		
Parasite prevalence microscopy [%] (CI95%)	55.4 (51.9-58.6)	30.7 (27.5-33.7)		
Parasite prevalence PCR [%] (CI95%)	68.7 (65.4-71.8)	37.9 (34.7-41.2)		
Number of symptomatic patients [%]**	13.2	3.2		

*Species analysis performed for subset of 201 patients.

**defined as microscopy positive and body temperature >37.5°C.

^1^by species-specific PCR.

^2^by drug resistance genotyping PCR.

### Parasite prevalence and density over time and comparing dry season and wet season

**Figure 1:**
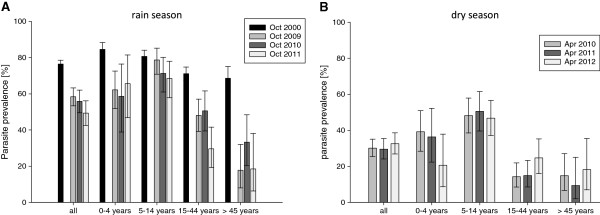
**Parasite prevalence stratified by age group based on microscopy.** Error bars represent 95% confidence intervals. **A**) Rainy season - baseline parasite prevalence from the rainy season of the year 2000 compared to parasite prevalence of 2009–2011; **B**) Dry season - parasite prevalence from 2010–2012.

Parasite prevalence was evaluated by microscopic examination of thin and thick blood smear at four time points at the end of the rainy season (October 2000, 2009, 2010 and 2011) and three time points at the end of the dry season (April 2010, 2011 and 2012). In the survey in October 2000, 78.9% of the individuals were parasite-positive by microscopy and 92.0% by PCR. The results obtained for the years from 2009 to 2012 indicate that the overall parasite prevalence has significantly decreased (Table 1 and Figure 1). In October 2009–2012 only 58.4%, 55.9%, and 49.3% respectively were positive by microscopy, which was supported by the estimates by PCR (92% in 2000 and 66.1%, 68.8% and 73.7% for 2009, 2010, and 2011 (p <0.001), see Table 1).

This effect was most pronounced for the highest age group (>45 years), where the prevalence for *P. falciparum* infections dropped from 85.1% in 2000 to an average of 23.2% by microscopy in 2009–2011 (see Figure 1). The effect was less pronounced in the age group five to 14 years, where parasite prevalence remained at around 69-80%.

In the dry season, between April 2010 and April 2012 (no baseline value from 2000 available) the parasite prevalence did not show marked variation with values between 29.6% and 32.6% by microscopy and 34.6% and 39.3% by PCR.

As expected the parasite prevalence for *Plasmodium* infections differed markedly between the hot dry season and the rainy season. The pooled estimate for the rainy seasons 2009–2011 was calculated at 55.4% (95% CI: 51.9-58.6%), as opposed to the pooled estimate from the dry seasons 2010–2012 with 30.6% (95% CI: 27.5-33.7%; Table 1). This variability between rain and dry season was even more pronounced with regard to parasite densities. In children below five years the geometric mean of parasite density was 2,124 parasites/μl (95% CI: 1,411-3,196) at the end of the rainy season (pooled data from 2009–2011) - compared to 360 parasites/μl (95% CI: 243–535) in the dry season. The same effect was also visible in the older age groups, although not as pronounced as in children below five years of age. The pooled estimates of geometric mean parasite density of rainy season *versus* the dry season exhibit a statistically significant difference for all age groups (Mann–Whitney U test, p-value <0.001).

### *Plasmodium* species distribution

**Figure 2:**
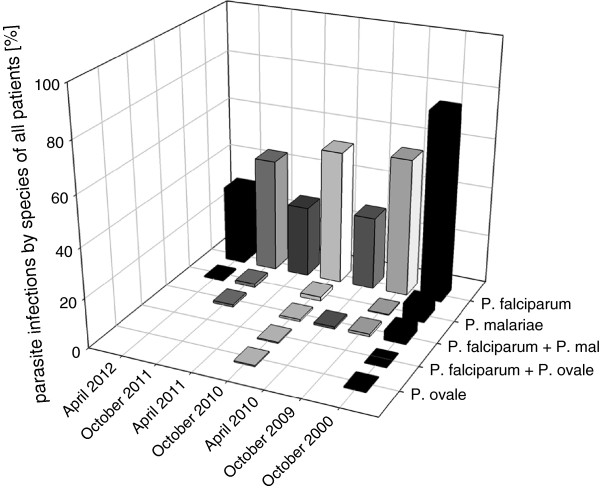
***Plasmodium *species distribution by year and season.** Time is on the x-axis, where April represents a sampling at the end of the dry season and October a sampling at the end of the rain season of the respective year. The y-axis shows the proportion of positive samples by microscopy. On the z-axis the different *Plasmodium* species are listed including co-infections.

Species-specific PCR, as well as microcopy, showed that *P. falciparum* is the most prevalent *Plasmodium* species (>95% of infections) in this region. Besides *P. falciparum, Plasmodium malariae* (2.7% of total infections) and *P. ovale* (0.3%) were detected in microscopy*.* Both these species were detected as single infections or co-infections with *P. falciparum* (Figure 2).

The distribution of malaria species differed between the rain and dry season. Infections or co-infections with *P. malariae* were less common in the dry season with 2.7% of microscopic detectable co-infections in 2010, none in 2011 and 1.2% single infections in 2012. The isolates from the rainy season contained 5.1% of *P. falciparum- P. malariae* co-infections in 2000, 1.7% in 2009, 1.4% in 2010 and 1.9% in 2011. Not only co-infections were more abundant, but also singular *P. malariae* could be found in the rainy season of 2000 and 2009–2011 (with 8.27%, 0.4%, 2.7% and 2.8% respectively). In total 85.7% of the *P. malariae* or *P. ovale* infections were found in children less than 15 years. Most of the non-falciparum infections had low parasites densities, so that only 23.8% of the isolates had counts of more than 1,000 parasites/μl.

Between 2000 to 2009–2012, this study shows a decreasing trend for *P. malariae* infections from 13.4% in 2000 to 2.1%, 4.1% and 4.7% in 2009–2011. Comparing the estimate from 2000 with the pooled estimate from 2009-2011 the t-test for proportions is statistically significant (p <0.001). *Plasmodium ovale* was only present in a small number of blood smears of October 2000 (0.7%) and 2010 (1.4%) (Figure 2).

### Asymptomatic malaria and frequency of fever

Asymptomatic malaria was defined as axillary body temperature <37.5°C at presentation (and no history of fever) with microscopically confirmed *Plasmodium* infection. In the rainy season, 85-90% of the parasite-positive patients were asymptomatic (85.3% in October 2009, 90.2% in October 2010 and 84.9% in October 2011), whereas in the dry season the number of asymptomatic individuals was higher at an average of about 97% (95.4% in April 2010, 98.7% in April 2011 and 96.2% in April 2012, see Table 1).

Symptomatic infections with high parasite density (>1,000 parasites/μl) were more likely to be observed in the rainy season, where 51.3% (59/115) of the symptomatic infections exceeded 1,000 parasites/μl (Table 2). During the dry season, only three of 16 symptomatic patients harboured more than 1,000 parasites/μl (18.7%) and all of them occurred in children between five and 14 years.

**Table 2:** Asymptomatic parasite carriers by parasite density (microscopy) and age group comparing rain and dry season

	**% asymptomatic carriers/total carriers**	**1-1,000 parasites/μl**	**1,000-2,500 parasites/μl**	**2,500-5,000 parasites/μl**	**5,000-10,000 parasites/μl**	**> 10,000 parasites/μl**
**Rainy season**						
**0-4 years**	68.1 (64/94)	80.6 (25/31)	61.5 (8/13)	75.0 (9/12)	76.5 (13/17)	42.9 (9/21)
(% asymptomatic carriers/total carriers)						
**5-14 years**	72.6 (170/234)	75.9 (101/133)	80.5 (33/41)	69.0 (20/29)	55.6 (10/18)	46.2 (6/13)
(% asymptomatic carriers/total carriers)						
**15-44 years**	86.4 (108/125)	86.8 (92/106)	81.8 (9/11)	100 (4/4)	(3/3)	(0/1)
(% asymptomatic carriers/total carriers)						
**>45 years**	86.2 (25/29)	84.6 (22/26)	(2/2)	(0/0)	(0/0)	(1/1)
(% asymptomatic carriers/total carriers)						
**All**	76.1 (367/482)	81.1 (240/296)	77.6 (52/67)	73.3 (33/45)	68.4 (26/38)	44.4 (16/36)
(% asymptomatic carriers/total carriers)						
**Dry season**						
**0-4 years**	92.5 (49/53)	90.9 (40/44)	(2/2)	100 (4/4)	(2/2)	(1/1)
(% asymptomatic carriers/total carriers)						
**5-14 years**	93.2 (136/146)	94.0 (109/116)	100 (17/17)	83.3 (5/6)	100 (4/4)	(1/3)
(% asymptomatic carriers/total carriers)						
**15-44 years**	96.3 (52/54)	96.0 (48/50)	(3/3)	(1/1)	(0/0)	(0/0)
(% asymptomatic carriers/total carriers)						
**>45 years**	100 (16/16)	100 (15/15)	(1/1)	(0/0)	(0/0)	(0/0)
(% asymptomatic carriers/total carriers)						
**All**	94.1 (253/269)	94.2 (212/225)	100 (23/23)	90.9 (10/11)	100 (6/6)	50 (2/4)
(% asymptomatic carriers/total carriers)						

The regression analysis revealed a significantly increased risk of fever in univariate logistic regression for patients in the rainy season as opposed to the dry season (p <0.001; see Table 3). Risk of fever in the univariate regressions was also associated with parasite prevalence, age and parasite density. Children between five and 14 years experienced a slightly increased risk of fever (not statistically significant) in the univariate regression compared to the reference (children 6 months to 4 years). However, in the age group between 15 and 45 years as well as the age group >45 years there was a significantly decreased risk of fever compared to the reference (by 73 and 86% respectively). Presence of cough as a proxy for upper respiratory infections was not significantly associated with presence of fever.

**Table 3:** Univariate and multivariate logistic regression analysis on presence of fever (axillary body temperature > 37.5°C) in 1,767 subjects participating in six surveys from 2009 to 2012 in Bourasso village, Nouna Health District, north-western Burkina Faso

	**Univariate**			**Multivariate**
	**No fever**	**Fever**	**OR (p-value)**	**OR (p-value)**
Season				
Wet season	69.9% (613/877)	30.1 (264/877)	Reference	reference
Dry season	88.8% (790/890)	11.2 (100/890)	0.29 (<0.001)	0.31 (<0.001)
Age groups*				
0–4 years	77.7% (240/309)	23.3% (69/309)	Reference	Reference
5–14 years	73.1% (457/625)	26.9% (168/625)	1.28 (0.13)	1.39 (0.06)
15–24 years	79.5% (186/234)	20.5% (48/234)	0.90 (0.61)	0.98 (0.94)
25–45 years	82.9% (295/356)	17.1% (61/356)	0.72 (0.09)	0.88 (0.53)
> 45 years	92.5% (221/239)	7.5% (18/239)	0.28 (<0.001)	0.31 (<0.001)
Parasite prevalence				
Slide positive [%] (total number)	73.7 (558/757)	26.3 (199/757)	1.83 (<0.001)	
Parasite density**				
no parasites	60.6% (849/1401)	45.2% (164/363)	Reference	Reference
1–500 parasites/μl	22.9% (321/1401)	24.8% (90/363)	1.45 (0.01)	1.00 (0.96)
501–1000 parasites/μl	5.9% (83/1401)	7.4% (27/363)	1.68 (0.028)	1.03 (0.90)
1001–2500 parasites/μl	4.6% (64/1401)	7.2% (26/363)	2.10 (0.003)	1.08 (0.77)
2501–5000 parasites/μl	2.9% (40/1401)	4.4% (16/363)	2.07 (0.018)	0.97 (0.92)
5001–10 000 parasites/μl	1.9% (26/1401)	5.0% (18/363)	3.58 (<0.001)	1.69 (0.12)
> 10 000 parasites/μl	1.3% (18/1401)	6.2% (22/363)	6.33 (<0.001)	3.08 (0.001)
cough	77.3% (136/176)	22.7 (40/176)	1.15 (0.462)	0.96 (0.85)

*Information on age missing in four patients.

** Information on parasite density missing in three patients.

In the multivariate regression the rainy season was still significantly associated with an increased risk of fever, adjusting for parasite density (categorical), age group, and cough. Other than dry season, high age and low parasite density are the best predictors for a reduced risk of fever, thus for being an asymptomatic parasite carrier. The OR for parasite density decreased in the multivariate regression compared to the univariate regression - probably due to adjusting for age as a correlate of acquired semi-immunity to malaria.

## Discussion

The data of this study showed a significant decline in parasite prevalence between the rainy season of 2000 and 2009–2011. A marked reduction of malaria incidence was described for countries in East Africa like Tanzania or Kenya [7-9,23]. However for Burkina Faso the WHO still reports rising case numbers, which could potentially be explained by increasing health service attendance and diagnosis of the disease [1]. Currently, one additional study from Nouna Health District in Burkina Faso confirms the observations of this study regarding a reduction of parasite prevalence from 85.8% to 65.5% during the rainy season and from 63.1% to 37.8% in the dry season from 1999 to 2009 in children less than four years of age. This study attributed the reduced parasite prevalence in children to the increased coverage of the population with insecticide-treated bed nets [24]. For the study that is reported here, there is no data that would allow estimating a possible mass effect of insecticide treated nets on the transmission intensity.

The reduced parasite prevalence over time was most pronounced in the group of children under five years of age - which might indeed be linked to the increased bed-net coverage. In children between five and 14 years of age the findings with regard to reduced parasite prevalence over time are not as pronounced, whereas the difference is significant again in older age. One hypothesis could be that this effect is associated with the age-specific role of immune mechanisms developing in populations exposed to malaria. The transmission decline if confirmed would also have an impact on acquisition of naturally acquired (semi-) immunity and consequently also ratio of symptomatic disease to asymptomatic disease [25,26]. Close monitoring provides important data that could help to improve malaria control also taking into account the experience of countries that have achieved similar transmission reductions.

### Mixed *Plasmodium* infections

The frequency of mixed infections with *P. malariae* decreased significantly during the study period from 2000 to 2009–2011. It was shown before in Malawi, that higher *P. falciparum* prevalence was linked with higher prevalence of minority species, which could be explained by the raised possibility of species co-transmission [27]. Furthermore, another study in Burkina Faso showed that the use of ITNs reduced the prevalence for *P. malariae* infections more substantially than that of *P. falciparum*[28]. As ITNs are now highly abundant in the study area, this could be another explanation for the different prevalence of *P. malariae* infections and co-infections between the baseline from the year 2000 and the results from 2009–2012.

The observation, that higher transmission is associated with greater species diversity, would also be in accordance to the results of this study for the dry season, which is a low transmission scenario. Prevalence for *P. malariae* is low in the April surveys with only three, zero or one infected patient for the years 2010–2012. These results are supported by similar findings of one study in Burkina Faso, near Bobo-Dioulasso, about 230 km south from the study site Nouna, where prevalence for *P. malariae* infections was reduced by approximately half in March and May compared to October and December [29]. However, previous findings in two other West African countries indicate that *P. malariae* exhibits opposing seasonal fluctuation with *P. falciparum*[30,31], which leads to increased prevalence of *P. malariae* during the dry season in those study areas.

Most of the infections and co-infections with *P. malariae* in this study showed low parasite density levels <1,000 parasites/μl. It is known that the blood stages of this *Plasmodium* species persist in the blood at low levels for long periods; some believe even lifelong [17,32]. This could potentially lead to an underestimation of the actual *P. malariae* burden, as the microscopy detection limit is low. These results demonstrate that PCR is a more sensitive tool to monitor the minor abundant *Plasmodium* species. Despite the use of molecular techniques, *P. ovale* infections in the study were still rare and limited to the high transmission time of the rainy season. Studies in children from Tanzania and Ivory Coast *P. malariae* were found to reduce fever and symptomatic disease [33,34]. The results obtained in this study show a trend, where in *P. malariae* and *P. falciparum* co-infections 30% of the patients were symptomatic, while in single *P. falciparum* 37.5% were symptomatic. However, the difference is not significant and the number of co-infected patients is low in the study.

### Asymptomatic parasitaemia

Asymptomatic parasitaemia was more frequent in the dry season even adjusting for parasite density and age group in a multivariate regression. Possible reasons for this observation include the existence of less pathogenic *P. falciparum* genotypes prevailing in the dry season, or the effect of a reduced incidence density during the dry season. The latter hypothesis would assume that clinical disease is also a cumulative result of a high number of sequential infections.

Evidence from Sudan suggests that chronic infections during the dry season are often multiclonal [35] and are competent to produce gametocytes [36]. It has also been reported that in patients with persistent *Plasmodium* infections during the dry season, a disease outbreak was associated with new genotypes, although it is not clear if this was a result of genetic crossing in the re-emerging *Anopheles* vector or if the genotype was imported from outside [37].

### Limitations

The age and sex distribution of the cross-sectional surveys is comparable over the years and also compared to the initial survey, which included the whole population of the village. The proportion of male participants was slightly reduced in some of the surveys, probably due to the fact that male inhabitants were busy with agricultural work. As prevalence and other epidemiological markers are not sex-specific this should not influence the results of this study.

## Conclusions

The results of this study suggest a decline of malaria transmission over the rainy seasons between 2000 and 2009–2011 in the region of Nouna, Burkina Faso. The parasite prevalence in the dry season was higher than expected with ~ 30% by microscopy throughout the years 2010–2012.

Asymptomatic parasitaemia was more frequent in the dry season even adjusting for parasite density and age group in a multivariate regression. Furthermore, the results of the study could show that reduced parasite prevalence for *P. falciparum* was associated with lower prevalence of *P. malariae* infections.

## Competing interests

The authors declare that they have no competing interests.

## Authors’ contributions

CG participated in the data collection, carried out laboratory investigations, analysed the data, produced figures and tables, and prepared the original draft. HKA carried out laboratory investigations and interpreted the data. GC, BC, and AS coordinated the data collection, contributed to the conception and design of the study, and interpreted the data. ML and HB analysed and interpreted the data. TJ conceived and coordinated the study, analysed the data, and prepared the original draft. All authors contributed to the drafts, read and approved the final manuscript.
